# Quantification of the methodological error in kinematic evaluation of the DRUJ using dynamic CT

**DOI:** 10.1038/s41598-023-29726-2

**Published:** 2023-02-23

**Authors:** J. G. M. Oonk, J. G. G. Dobbe, S. D. Strackee, G. J. Strijkers, G. J. Streekstra

**Affiliations:** 1 Department of Biomedical Engineering and Physics, Amsterdam UMC, Meibergdreef 9, 1105 AZ Amsterdam, The Netherlands; 2 Department of Plastic-, Reconstructive- and Handsurgery, Amsterdam UMC, Meibergdreef 9, 1105 AZ Amsterdam, The Netherlands; 3 Amsterdam Movement Sciences, Musculoskeletal Health, Restoration and Development, Amsterdam, The Netherlands

**Keywords:** Bone, Biomedical engineering, Computed tomography

## Abstract

Distal radio-ulnar joint (DRUJ) motion analysis using dynamic CT is gaining popularity. Following scanning and segmentation, 3D bone models are registered to (4D-)CT target frames. Imaging errors like low signal-to-noise ratio (SNR), limited Z-coverage and motion artefacts influence registration, causing misinterpretation of joint motion. This necessitates quantification of the methodological error. A cadaver arm and dynamic phantom were subjected to multiple 4D-CT scans, while varying tube charge-time product and phantom angular velocity, to evaluate the effects of SNR and motion artefacts on registration accuracy and precision. 4D-CT Z-coverage is limited by the scanner. To quantify the effects of different Z-coverages on registration accuracy and precision, 4D-CT was simulated by acquiring multiple spiral 3D-CT scans of the cadaver arm. Z-coverage was varied by clipping the 3D bone models prior to registration. The radius position relative to the ulna was obtained from the segmentation image. Apparent relative displacement seen in the target images is caused by registration errors. Worst-case translations were 0.45, 0.08 and 1.1 mm for SNR-, Z-coverage- and motion-related errors respectively. Worst-case rotations were 0.41, 0.13 and 6.0 degrees. This study showed that quantification of the methodological error enables composition of accurate and precise DRUJ motion scanning protocols.

## Introduction

Four-dimensional computed tomography (4D-CT) is emerging as a valuable technique in the study of healthy and pathological distal radio-ulnar joint (DRUJ) function^1–3^. Scanning and subsequent image analysis result in a 3D representation of the joint in motion. The dynamic 3D model provides additional information on motion-related pathology, like osseous impingement or instability, which is lacking in conventional static 3D imaging^4^. Apart from the improvement in the diagnosis of motion-related pathologies, the 4D-CT frames can be used to quantify DRUJ kinematics. With this information, a comparison between motion patterns of the pathological and healthy contralateral joint can be made, which, for example, can be used to design a patient-specific prosthesis that optimally restores DRUJ movement.

An error in 4D-CT imaging directly impacts visualization and quantification of joint motion which could lead to a wrong conclusion or diagnosis. Therefore, the methodological error should be taken into consideration when using 4D-CT quantitatively. Although the use of 4D-CT is increasing, reports on the methodological errors are limited.

In 4D-CT analysis of the distal radio-ulnar joint (DRUJ), the main sources of error are a low signal-to-noise ratio (SNR), limited Z-coverage, and motion artifacts. The SNR denotes the ratio of the amplitude of the desired signal to that of the unwanted noise^5^.Z-coverage refers to the extent of image data acquired along the z-axis^6^ and motion artifacts are distortions or errors in the image due to patient movement during the scan^7^.These errors are dependent on the scanning protocol and the scanner used and could negatively affect image registration, which is an important step in 4D-CT motion analysis. During image registration, segmented bone models are aligned with subsequent 4D-CT target frames. Registration depends heavily on correspondence between grayscale values of a bone in the 3D-CT source image and the target 4D-CT frames^8^. Quantum mottle, the most common form of noise, is invariant. Therefore, the SNR is dependent on the amount of signal (mAs) provided by the CT-scanner. Low SNR and motion artefacts can affect local image intensity levels, which may lead to an error in registration. A small Z-coverage in 4D-CT target frames in comparison to the 3D-CT source image could influence registration as well since the 4D-CT target frames contain fewer distinct features for registration.

This study aimed to quantify the effects of low SNR, limited Z-coverage and motion artefacts in 4D-CT on the registration errors in the analysis of DRUJ motion.

## Methods

### Cadaver specimen and image acquisition

A cadaver study was conducted as an alternative to using CT scans on volunteers, as the number of CT scans required to evaluate all experimental conditions would result in unacceptable levels of radiation exposure for healthy individuals. This study was approved by the medical ethical comittee of the Amsterdam UMC (ref: W22_226, #22.325), informed consent was obtained from the donor and all experiments described below were conducted in accordance with the relevant guidelines and regulations.

One fresh frozen cadaver upper limb, resected at the trans-humeral level, was thawed for 24 hours before scanning. An initial CT scan of the arm did not show any signs of trauma or osteoarthritis.

4D- as well as 3D-scanning, was performed using a SOMATOM Force CT-scanner (Siemens Healthineers AG, Erlangen, Germany). All 4D-CT scans were made using a single source protocol developed in collaboration with Siemens. This protocol utilized continuous scanning at maximum gantry velocity similar to fluoroscopy to be able to achieve high temporal resolution. For 3D- as well as 4D-CT acquisitions the tube voltage remained constant at 120 kVp. Further details on the imaging protocols can be found in table 1.

**Figure 1:**
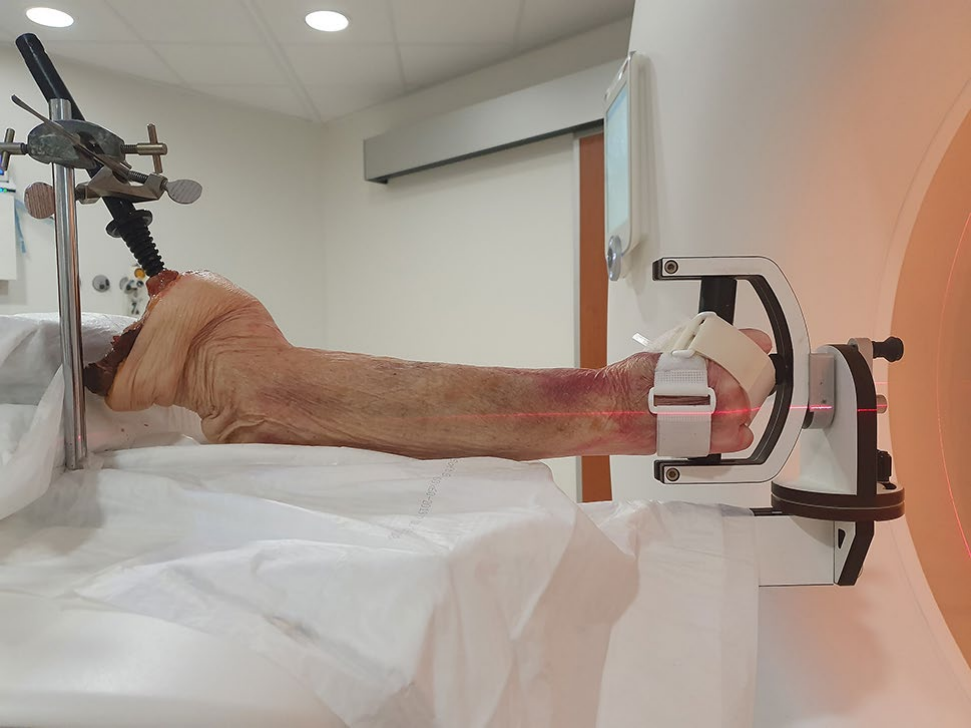
Scanning setup: Cadaveric sample mounted in the hand guiding device.

To obtain images with different Z-coverage- and SNR levels, the arm was placed in a custom-made hand guiding device (Fig. 1) with the wrist in the neutral position between pronation and supination. Fixation of the arm was achieved through use of an intramedullary pin inserted in the humerus and Velcro straps securing the hand to the hand-grip (Fig 1). For the purpose of obtaining images with different Z-coverages, the 4D-CT scans were replaced by a series of spiral CT scans as the used CT scanner was only capable of 4D-CT scans with a z-coverage of 57 mm. The entire forearm was scanned ten times, one source image and nine target images, at a tube current-time product level of 120 mAs. Beam pitch was set to 0.8 such that there was no need for interpolation during registration as a result of gaps in the projection data, minimizing spiral artifacts in the reconstructed images. Prior to registration to these images, the radius and ulna surface meshes resulting from segmentation were clipped to four specific sizes, each simulating a different Z-coverage. For the evaluation of SNR, three spiral CT source images as well as three 4D-CT scans (33 frames each) were made at 30-, 75- and 120 mAs. A higher tube current-time product equates to a higher SNR^9^ and thus a relative decrease in image noise.

**Figure 2:**
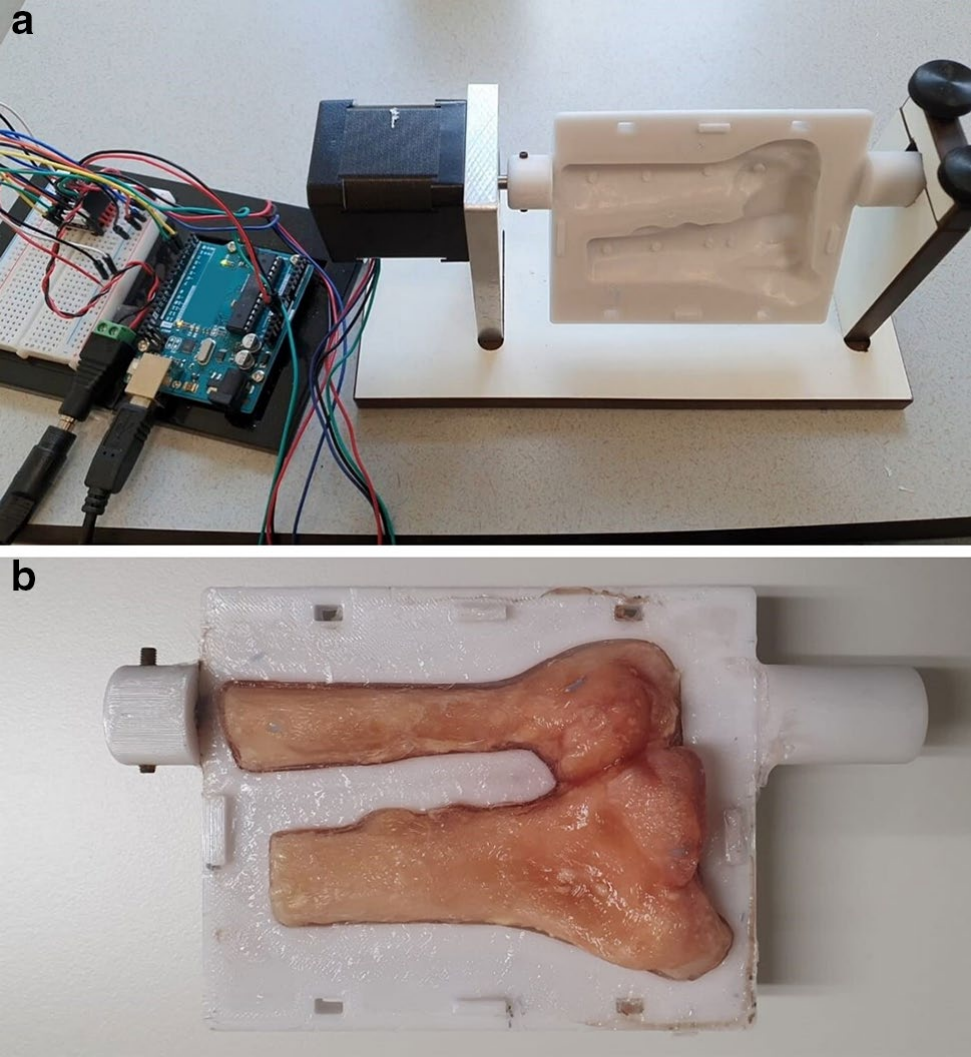
Motorized phantom: A: The wrist phantom attached to the computer controlled stepper motor with the rotation axis in line with the ulnar shaft. B: Wrist phantom with the DRUJ embedded in agarose gel.

To investigate motion artefacts, an in-house developed DRUJ phantom was used which could rotate the distal radius and ulna at various angular velocities using a stepper motor. A phantom was chosen as it could provide a precisely controlled angular speed. A simplified wrist model was designed using CAD software. The simulated wrist had an elliptical cross-section and a cavity in which the distal radius and ulna of the cadaver specimen were placed before performing the experimental evaluations. The axis of rotation was placed at the centre of the ulnar shaft (Fig 2a). This ensured that the ulna would not be severely affected by motion artefacts, while the maximum motion artefacts would occur at the lateral side of the radius, similar to what can be expected for in vivo scans.

**Figure 3:**
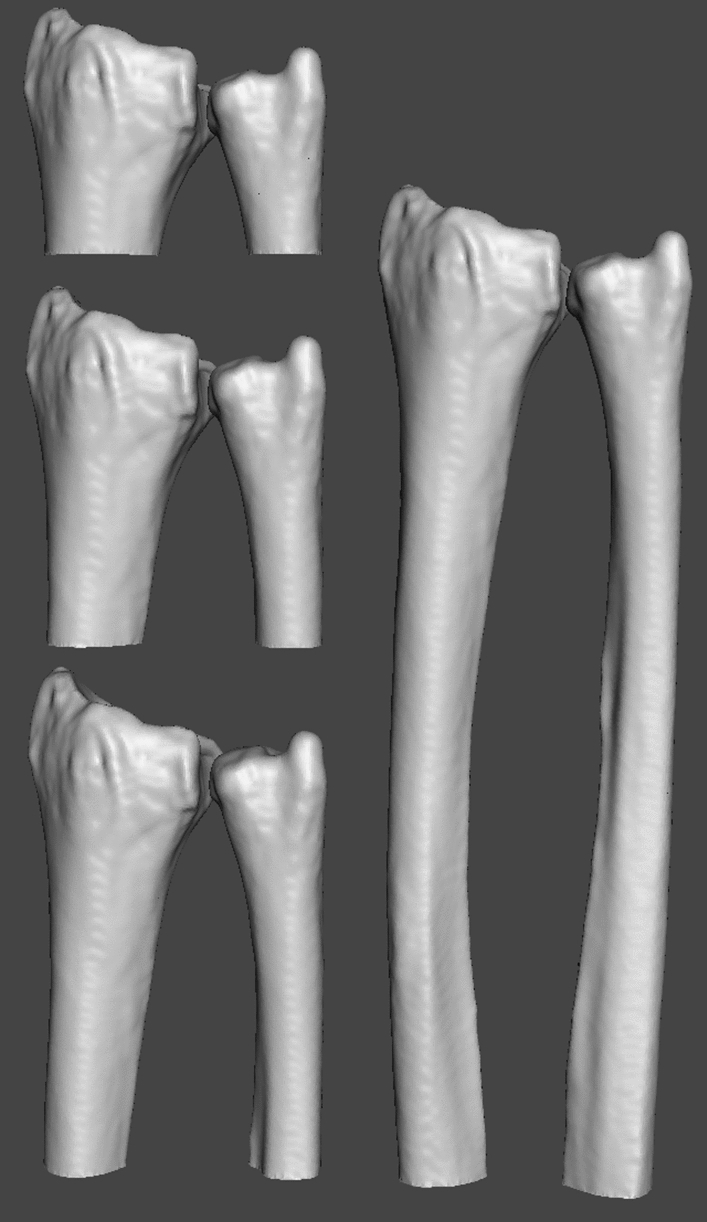
Radius and ulna 3D-model size at different Z-coverages: The 3D-models of the radius and ulna used in registration at four different Z-coverage levels. The cutting plane for the 3D-models was defined using a bounding box on the radius. The height of the bounding box was determined by the desired Z-coverage. The bottom plane of the bounding box was used to clip the 3D-model of the radius as well as the ulna.

The distal radius and ulna were excised from the arm by an experienced hand surgeon (S.S.), leaving the DRUJ and triangular fibrocartilage complex (TFCC) intact to maintain the original spacing between the radius and ulna. The radius and ulna were fixated within the wrist model by pouring agarose gel (2% w/v) into the cavity between the bones and the wrist model (Fig. 2b). Apart from fixation, this also provided a similar CT image contrast between the bone cortex and the surrounding agar compared to the in vivo situation.

Scanning consisted of one spiral CT for segmentation purposes and seven 4D-CT scans at increasing angular velocity ranging from 1 to 30 degrees per second. Each 4D-CT scan contained 33 image frames. The tube current-time product was set to a relatively high level of 120 mAs to maximize the SNR and thus isolate the effect of motion artefacts.

**Table 1:** Key imaging protocol details. Bold font indicates the clinical effective dose in spiral 3D-CT and 4D-CT (33 frames).

Variables	Protocol		
	spiral 3D-CT	4D-CT	Clinical hand/wrist exam
Tube current-time product	30/75/120 mAs	120 mAs	Automated current adjustment
Slice thickness	0.6 mm	0.6 mm	0.6 mm
In plane resolution	512x512	512x512	512x512
Rotation time	0.25 s	0.25 s	Scanner dependent
Reconstruction kernel	Br64s	Br64s	Br64s
Effective dose	**6.7** $\varvec{\mu Sv}$ (30 mAs) 16.7 $\mu Sv$ (75 mAs) 26.8 $\mu Sv$ (120 mAs)	**26.4 ** $\varvec{\mu Sv}$ (30 mAs) 1.05 mSv (120 mAs)	8.9 $\mu Sv$ (80 mAs)^10^
Z-coverage	50 cm	57 mm	25 cm

### Image analysis

DICOM images were imported into custom made software developed at Amsterdam UMC in the Netherlands^8^. The bones were segmented from the CT image and subsequently registered to each of the 4D-CT or spiral CT target frames, to obtain their corresponding transformation matrices. The transformation matrices were then used to quantify the apparent displacement between the radius and ulna, which represented the error in registration.

The radius and ulna were segmented by a threshold-connected region growing algorithm, starting at a seed-point location indicated by the user. A binary filling operation was used to close residual holes^11^. The result thus obtained served to initialize a Laplacian level-set growth algorithm, which advances voxel dispersion toward the edge of the bone^12^. Finally, a surface mesh was extracted at the zero level of the level-set image, using the marching cubes algorithm^13^.

In this study, image-to-image registration was used to transform the radius and ulna surface meshes to the correct orientation and position in the target images. At first, a double contour surface mesh was generated from the initial segmentation surface mesh by expanding and reducing the surface along the surface normal. The source image was sub-sampled at the point locations of the double contour surface mesh creating a subset of the source image voxels containing the distinct border between soft tissue and bone cortex. The subsampling aids in accelerating the registration process without compromising on accuracy, as the most distinctive features of the image are maintained. Following a rough manual repositioning of the segmentation surface mesh to the target frame, the registration was optimized^8^ through a comparison of the pixel grey values in the subset of the source- and the target images.

#### SNR and Z-coverage

Segmentation of the entire radius and ulna was performed in the dedicated spiral CT source image. The surface meshes resulting from the SNR source images were then directly registered to the 4D-CT target images with the corresponding mAs level. For the analysis of Z-coverage the first scan in the series of 10 spiral 3D-CT scans was used for segmentation purposes and the remaining nine were used for registration.

The registration error due to the limited Z-coverage was investigated by clipping the radius and ulna surface meshes to different lengths prior to registration to mimic a decreased Z-coverage. The tested surface mesh lengths were 40-, 57-, 80- and 160 mm (fig. 3), representing the approximate range of Z-coverage in current 4D-CT capable scanners (e.g. GE Discovery CT750 HD, Siemens Somatom Force (used in this study), Phillips Brilliance iCT and Toshiba Aquilion ONE respectively). The clipped surface meshes were only registered to the series of 120 mAs simulated 4D-CT target images to minimize the influence of noise during registration.

#### Motion artefacts

The registration error caused by motion artefacts was investigated by segmentation of the radius and ulna surface meshes from the 3D-CT source image and subsequent registration to the target images in a 4D-CT scan, made at the seven different angular velocities. As described in the image analysis paragraph, a manual registration was performed prior to running the registration algorithm. At lower angular velocities the manual registration only needed to be performed once, in the first frame, as the displacement of the radius and ulna outlines between target frames was small enough to provide sufficient overlap of the surface mesh outline and the corresponding bone in the target image for the optimization algorithm to automatically register to the new position.

#### Error parameters

In both cadaveric experiments, the radius remained static with respect to the ulna. Although this approach does not induce physiological motion of the radius about the ulna, it does enable evaluating the reliability of kinematic DRUJ analysis, since the segmented position of the radius with respect to the ulna serves as gold standard position. Any apparent motion that is observed between the radius and the ulna in the rotating phantom, can therefore be ascribed to motion analysis (i.e. registration errors), and can be affected by SNR, z-coverage or motion. With regard to the latter, the phantom was designed in such a way that the center of rotation at the level of the DRUJ was equal to the center of rotation found in literature regarding DRUJ motion^14,15^. This results in motion artefacts at the edge of the radius, being farther away from the axis of rotation, while limiting motion artefacts at the ulna which is similar to the physiological situation. With the use of the relative position of the radius and ulna as reference, any apparent movement of the radius with respect to the ulna after image analysis would indicate an error in registration due to a reduced Z-coverage, low SNR or motion artefacts. The translation vector for each target frame, 9 for Z-coverage and 33 for SNR and motion artefacts, was extracted directly from the transformation matrix. The rotation vectors were calculated from each target frame’s rotation matrix using the Tait-Bryan convention and the YXZ sequence. The resulting 3x1 translation- and rotation vectors were further condensed by calculation of the vector magnitude. The magnitudes were chosen as error parameters in accordance with previously published work^8^ and robotic literature^16^. Visualization of the errors was done using box plots, showing the median, interquartile range (IQR), maximum and minimum values and outliers. Accuracy of registration is represented by the median value of the translational- and rotational error of a specific protocol. Precision is defined by the variability of the error. The variability of the error also provides a characterization of reproducibility as, in the case of SNR and Z-coverage, registration is performed on virtually the same target frame multiple times.

### Statistics

For the statistical analysis, Python 3.9 in combination with a statistical function package, scipy.stats v1.7.1, was used. Each group of vector magnitudes represented a sample. A Shapiro-Wilk normality test was used to analyze the SNR and motion artefact samples. Z-coverage samples were assumed to be non-parametric due to their small size. Differences in precision, indicated by variance between experimental evaluations, were established using the ansari-bradley test for SNR- and Z-coverage samples and the Welch T-test (two-tailed) for the motion artefact samples. As the ansari-bradley test can produce misleading results if the medians of both samples are not equal, the samples were first normalized around zero (by subtracting the sample median). The accuracy, indicated by the median, was tested with a Kruskall-Wallis test for samples with equal variance and a Mood’s median test for the other samples. An additional linear regression for the motion artefact samples was performed to evaluate the relations between the angular velocity and the accuracy and precision. P-values related to translation errors and p-values related rotation errors were reported as p_t_ and p_r_ respectively. The level of significance was set at 0.05.

## Results

### SNR

**Figure 4:**
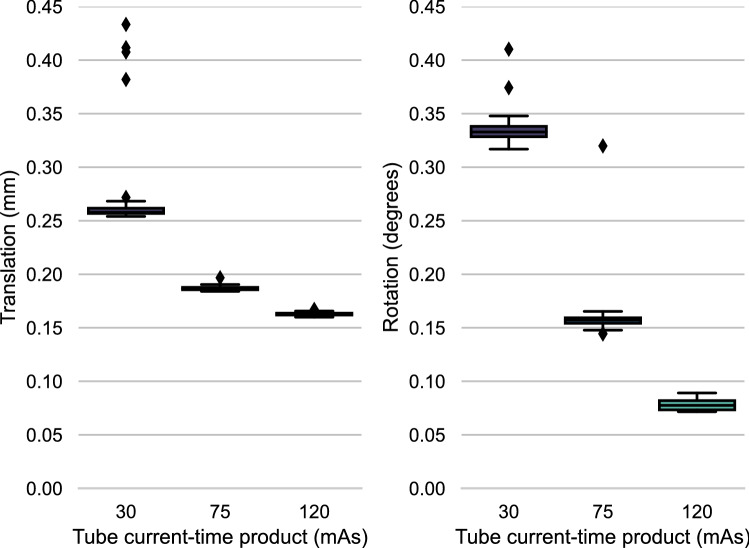
SNR induced error: The error in registration at different levels of tube current-time product, leading to different SNR’s. The translational error in mm is shown in the left subgraph, while the rotational error in degrees is given by the right subgraph. All boxplots in this study show the median (horizontal line), interquartile range (IQR) (box), values within the range Q1-1.5*IQR to Q3+1.5*IQR (whiskers) and values outside of the previous range (outliers). Imaging technique: 4D-CT.

The registration error with different SNR’s is shown in Fig. 4. In general, the rotational and translational errors were smaller than 0.8 degrees and 0.4 mm, respectively. The Shapiro-Wilk test determined the samples were non-parametric. Rotational as well as translational accuracy improved significantly (p_t_ $< 0.001$, evaluated with Mood’s median test) with every rise in tube current-time product level. Furthermore, translational- and rotational precision increased when the tube current-time product was increased from 30- to 75 mAs. With p_t_=0.027 for the translational precision and p_r_=0.012 for the rotational precision. Other changes were found to be insignificant.

### Z-coverage

**Figure 5:**
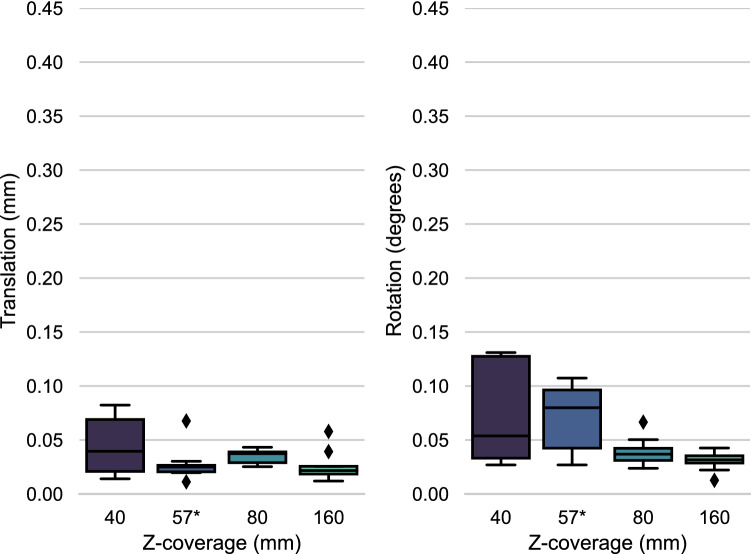
Z-coverage induced error: The error in registration at different levels of Z-coverage. The translational error in mm is shown in the left subgraph, while the rotational error in degrees is given in the right subgraph. Imaging technique: serial spiral 3D-CT. *= Z-coverage of scanner used in this study.

The error with increasing Z-coverage is depicted in Fig. 5. The maximum translational- and rotational errors, 0.08 mm and 0.13 degrees respectively, were smaller than the SNR-related errors. With the increase from 57- to 80 mm Z-coverage the rotational accuracy improved significantly (p_r_=0.038, evaluated with the Kruskall Wallis test), no notable change was observed in the translational accuracy. Similar to the translational accuracy, translational precision did not improve by increasing the Z-coverage. Rotational precision did improve from 40- to 80 mm (p_r_=0.026).

**Figure 6:**
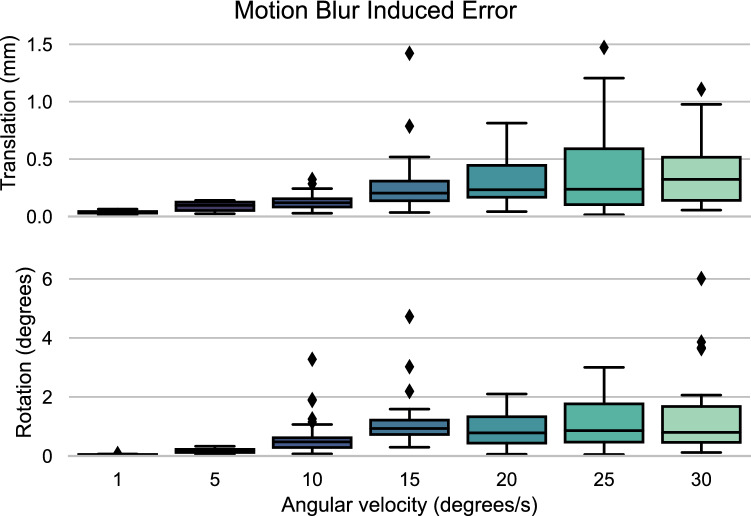
Motion artefact induced error: The error in registration when the scanned subject is rotating at different angular velocities. The translational error in mm is shown in the upper subgraph, while the roational error in degrees is given by the lower subgraph. Imaging technique: 4D-CT.

**Figure 7:**
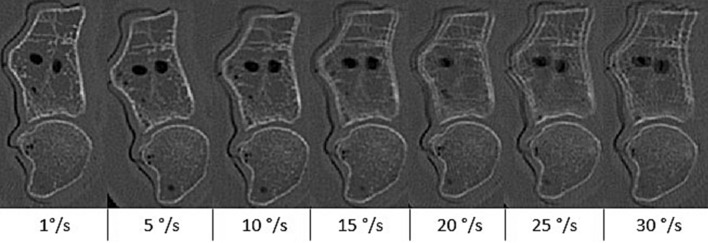
Target frame motion artefacts: CT target images at varying angular velocities. Note the shift from a blurring to a ghosting artefact at speeds greater than ten degrees per second.

### Motion artefacts

The translational and rotational errors due to motion artefacts, showed in Fig. 6, had a maximum of 1.5 mm and 6 degrees. The Shapiro-Wilk test showed the samples were normally distributed. Translational- and rotational accuracy decreased significantly from 1- to 5- (p_t_ and p_r_$$< 0.001)$$ and 10- to 15 degrees per second(p_t_ = 0.024, p_r_ $$<0.001)$$. Additionally, rotational accuracy decreased from 5- to 10 degrees per second as well (p_r_ $$<0.001)$$. Translational- and rotational precision also deteriorated notably when increasing the angular velocity from 1- to 5- (p_t_ and p_r_ $$<0.001)$$, 5- to 10- (p_t_ = 0.009, p_r_ $$<0.001$$) and 10- to 15 degrees per second (p_t_ = 0.005, p_r_ = 0.009). The linear regression showed a positive correlation between the decrease in translational- and rotational accuracy and precision and the increase in angular velocity (Accuracy: p_t_$$<0.001$$, p_r_ = 0.014. Precision: p_t_ = 0.029, p_r_ = 0.034. all R-values $$> 0.79)$$.

## Discussion

This study evaluated the effect of SNR, Z-coverage and motion artefacts on registration accuracy in motion analysis of the DRUJ using 4D-CT. Inaccurate registration leads to errors in the visualization of DRUJ movement and quantification of kinematic parameters.

Different imaging modalities have been used to perform DRUJ motion analysis^15,17,18^. However, the effects of methodological errors on the DRUJ motion analysis are often neglected^15,17^, or extrapolated from errors measured for different joints or scanning protocols^18^. This may lead to inconclusive results when the magnitude of the methodological error is close to, or larger than the magnitude of the measured kinematic parameter. Moreover, it is unlikely that registration errors are the same for different joints as one joint can have more distinct features in comparison to another joint, which can improve registration accuracy and precision.

Several studies reported on the methodological errors for various imaging techniques used to study the kinematics of the knee, wrist and glenohumeral joint^19–22^. The translational errors for MRI combined with fluoroscopy of the knee (tibia and femur) using a scanning plane based coordinate system and measurement along the SI and ML axis, were [0.74 2.0 mm]^19^. For MRI combined with biplane fluoroscopy imaging of the glenohumeral joint (humerus), using a humerus based coordinate system, translations were [1.0 1.0 1.6 mm]. The CT to (biplane) fluoroscopy translational error, in the analysis of the knee and the glenohumeral joint, were [0.53 1.6]^21^ and [0.8 0.8 0.9]^20^ mm. For CT-4D-CT, as used in this paper, translational errors were found to be [0.10 0.14 0.11]^19^ and [0.028 0.041 0.132]^22^ mm for the knee and wrist joint (radius), respectively. The former study used a humerus- and tibia based coordinate system while the latter study used a scanner based coordinate system. It has to be noted that the errors found in registration to fluoroscopy are the “in-plane” errors, as fluoroscopy is a 2D image modality. With 2D imaging out-of-plane errors become bigger than in-plane errors as 3D information is absent. This indicates that the methodological error in 4D-CT, irrespective of the imaged joint, is relatively small compared to other joint motion analysis techniques.

This study also uses two imaging modalities. Namely 4D-CT and a series of spiral 3D-CT. The latter was used to overcome the issue of the limited Z-coverage in the scanner used for the experiments. As the Z-coverage results originate from a different imaging modality, the absolute errors can not be translated directly to 4D-CT. However, it is likely that the observed trend, an increase in translational and rotational precision, will apply to 4D-CT data as well. The differences between the series of spiral 3D-CT and 4D-CT with the equal Z-coverage of 57 mm can be found by comparing the 120 mAs results in Fig. 4 with the 57 mm results in Fig. 5. It shows slightly worse translational accuracy for 4D-CT but similar rotational accuracy. Translational- and rotational precision improve in 4D-CT with respect to a series of spiral 3D-CT scans. These results in addition to the lack of spiral artefacts, lack of scanner table movement and a shorter scanning time advocate for the use of 4D-CT. Finally, one can argue that a series of spiral CT scans does not really visualize movement as the joint of interest is visualized in static positions along the range of motion and thus not actually moving during the scan.

The rotational and translational errors in this study showed a similar trend in the analysis of SNR and Z-coverage. With the increase of tube current-time product and Z-coverage, significant improvements in registration accuracy and precision were observed. The positive effect on registration caused by an increased tube current time product can be explained by a relative decrease in image noise, i.e. an increased SNR, with fewer discrepancies in corresponding voxel intensities. The increase in Z-coverage provided increased mutual information between the source- and target images, thus improving registration^23,24^. The effect of an increase in Z-coverage may be more pronounced when imaging different joints, such as the ankle joint, as they may contain more distinct features than the radial- and ulnar shaft as used in this study.

An increase in motion artefacts associated with an increase in angular velocity led to a higher registration error. As displayed in Fig. 7, motion blur transitioned into a ghosting artefact at angular velocities of ten degrees per second or higher. The presence of two contours in the target frame makes it difficult for the operator and the registration algorithm, during manual and automated registration respectively, to unambiguously select the correct contour, assuming a correct contour is even present in the image. This leads to the observed increase in the spread of the error and thus a deteriorated precision. As the overlap between the original- and ghost contour decreases with increasing angular velocity, the registration algorithm might develop a preference to register to one contour more than the other. This could explain the slight increase in the precision at an angular velocity of 30 degrees per second. In general, the effect of the motion artefact induced error turns out to be more substantial than the error caused by a low SNR or limited Z-coverage.

At first sight, the measured registration errors could be considered small or even negligible. However, it is important to realize that a small error in registration at the distal forearm could lead to a large error at the proximal forearm. For instance, when scanning a male patient with an average radius length of 26.5 cm^25^ with a 4D-CT, 40 mm Z-coverage, scanning protocol, a 1-degree rotational error at the proximal radius leads to a translational error of 4.6 mm $(tan(1)*26.5)$ at the distal end of the radius. These errors can be of great influence in quantifying kinematic parameters as the order of magnitude of the kinematic parameters is similar to that of the errors^18,26^.It emphasizes the need for a carefully considered scanning protocol and warrants the acquisition of an additional scan, of a different area of the forearm, if needed.

Study limitations include the use of serial spiral CT instead of 4D-CT in the analysis of  Z-coverage. Unfortunately, an optimal, large, Z-coverage could not be achieved, as the scanner used was limited to a Z-coverage of 57 mm. In the analysis of Z-coverage, the use of a serial spiral CT scan was therefore required to simulate the larger Z-coverages ( $>57$ mm). To maintain consistency in the analysis of the Z-coverage variable, the small Z-coverage of 40 mm was also simulated using a serial spiral CT. As mentioned before, we found that for 57 mm Z-coverage, registration accuracy was similar between 4D-CT and a series of spiral CT while precision of registration improved when using 4D-CT over serial spiral CT.

Another limitation was that the reported errors are specific to the image-to-image registration method used in this study. Other registration algorithms, like iterative closest point (ICP) registration algorithms^25,27^, are based on a different registration technique. ICP requires multiple segmentations, which introduces a different error source leading to different registration accuracy and precision.

The sample size of 1 could also be viewed as a limitation since the presence of more defined geometric bony landmarks could improve registration while bones with a smoother geometry would likely worsen registration quality. Even though the registration process is impacted by geometric factors, it is not expected to result in vastly different errors than those reported in this study.

As motion artefacts were found to have the largest impact on registration accuracy, it is recommended to carefully consider the motion speed of the subject as the determining factor in a scanning protocol. The biggest gains in registration accuracy and precision can be achieved by slow movement of the joint or by using a very sensitive scanner. As the motion speed during scanning is related to scanning time, it will directly influence patient radiation dosage and should be managed by tuning tube current-time product. A scanner with a large Z-coverage is preferable. However, other scanner properties like maximum gantry velocity and a single- or dual source setup should be taken into consideration as well. These properties influence the maximum temporal resolution which, in turn, influences the formation of motion artefacts.

Using the results described in this study, a suitable scanning protocol for joint motion analysis for the DRUJ can be designed. Future studies should focus on the quantification of the kinematic parameters. Even though this study advises on a slow DRUJ movement to reduce the negative effect of motion artefacts on registration, faster movement should still be analysed as the speed could also have an effect on the motion pattern. This also applies to use of different elbow angles^28^ and resistive forces^15^. A better understanding of DRUJ kinematics can then be used for multiple applications ranging from improved physical therapy to arthroplasty procedures.

## Conclusion

This study successfully quantified the effect of SNR, reduced Z-coverage and motion artefacts on registration accuracy and precision in the kinematic CT analysis of DRUJ motion.The influence of motion artefacts was found to have the greatest impact on the registration process during image processing. The results of this study help to predict error margins prior to scanning. This enables researchers and radiologists to adapt the scanning protocol such that the correct balance between radiation dosage and a low registration error can be achieved with the available hardware. The 4D-CT scans resulting from these protocols can be used in the quantitative analysis of DRUJ motion.

## Supplementary Information

Supplementary Information.

## Data Availability

All data generated or analysed during this study are included in this published article (and its Supplementary Information files).

## References

[1] Shakoor D (2019). Kinematic analysis of the distal radioulnar joint in asymptomatic wrists using 4-dimensional computed tomography-motion pattern and interreader reliability. J. Comput. Assist. Tomogr..

[2] Shores, J. T., Demehri, S. & Chhabra, A. Kinematic, “4 Dimensional” CT Imaging in the Assessment of Wrist Biomechanics Before and After Surgical Repair. *Eplasty* **13**, e9 (2013). PMC3589877 23573338

[3] Choi YS (2013). Four-dimensional real-time cine images of wrist joint kinematics using dual source CT with minimal time increment scanning. Yonsei Med. J..

[4] Carr R, MacLean S, Slavotinek J, Bain G (2019). Four-dimensional computed tomography scanning for dynamic wrist disorders: Prospective analysis and recommendations for clinical utility. J. Wrist Surg..

[5] Verdun, F. R. *et al.**Image quality in CT: From physical measurements to model observers*10.1016/j.ejmp.2015.08.007 (2015). 10.1016/j.ejmp.2015.08.007 26459319

[6] Hu H (1999). Multi-slice helical CT: Scan and reconstruction. Med. Phys..

[7] Gondim Teixeira, P. A. *et al.* Evidence-based recommendations for musculoskeletal kinematic 4D-CT studies using wide area-detector scanners: a phantom study with cadaveric correlation. *European Radiology* **27**, 437–446, 10.1007/s00330-016-4362-y (2017). 10.1007/s00330-016-4362-y 27095320

[8] Dobbe JG, De Roo MG, Visschers JC, Strackee SD, Streekstra GJ (2019). Evaluation of a quantitative method for carpal motion analysis using clinical 3-D and 4-D CT protocols. IEEE Trans. Med. Imaging.

[9] Sprawls, P. AAPM tutorial. CT image detail and noise. *Radiographics : a review publication of the Radiological Society of North America, Inc* **12**, 1041–1046, 10.1148/radiographics.12.5.1529128 (1992). 10.1148/radiographics.12.5.1529128 1529128

[10] Ludlow JB, Johnson BK, Ivanovic M (2018). Estimation of effective doses from MDCT and CBCT imaging of extremities. J. Radiol. Prot..

[11] Carelsen B (2009). Detection of in vivo dynamic 3-D motion patterns in the wrist joint. IEEE Trans. Biomed. Eng..

[12] Ibanez, L. & Schroeder, W. The Insight Segmentation and Registration Toolkit, Software Guide. *The ITK Software GUIDE* 805 (2014).

[13] Lorensen, W. E. & Cline, H. E. Marching cubes: A high resolution 3D surface construction algorithm. *Proceedings of the 14th Annual Conference on Computer Graphics and Interactive Techniques, SIGGRAPH 1987* **21**, 163–169, 10.1145/37401.37422 (1987).

[14] Oki S (2019). The relationship between the morphological axis and the kinematic axis of the proximal radius. Surg. Radiol. Anat..

[15] Tay SC (2008). A method for in-vivo kinematic analysis of the forearm. J. Biomech..

[16] Kuo, H.-Y., Su, H.-R., Lai, S.-H. & Wu, C.-C. 3D object detection and pose estimation from depth image for robotic bin picking. In *2014 IEEE International Conference on Automation Science and Engineering (CASE)*, August, 1264–1269, 10.1109/CoASE.2014.6899489 (IEEE, 2014).

[17] Nakamura T, Yabe Y, Horiuchi Y, Yamazaki N (1999). In vivo motion analysis of forearm rotation utilizing magnetic resonance imaging. Clin. Biomech..

[18] Matsuki KO (2010). In vivo 3D kinematics of normal forearms: Analysis of dynamic forearm rotation. Clin. Biomech..

[19] Moro-oka T-A (2007). Can magnetic resonance imaging-derived bone models be used for accurate motion measurement with single-plane three-dimensional shape registration?. J. Orthop. Res..

[20] Akbari-Shandiz M (2019). MRI vs CT-based 2D–3D auto-registration accuracy for quantifying shoulder motion using biplane video-radiography. J. Biomech..

[21] Oki S (2019). Four-dimensional ct analysis using sequential 3d–3d registration. J. Vis. Exp..

[22] Zhao K (2015). A technique for quantifying wrist motion using four-dimensional computed tomography: Approach and validation. J. Biomech. Eng..

[23] Ofverstedt, J., Lindblad, J. & Sladoje, N. Fast and Robust Symmetric Image Registration Based on Distances Combining Intensity and Spatial Information. *IEEE Transactions on Image Processing* **28**, 3584–3597, 10.1109/TIP.2019.2899947 (2019). 1807.11599. 10.1109/TIP.2019.2899947 30794174

[24] Pennec, X., Ayache, N., Landmark-based, J.-p. T. & Thirion, J.-p. Landmark-based registration using features identified through differential geometry. In *Handbook of Medical Imaging - Processing and Analysis.*, pp.499–513 (2011).

[25] Mall G (2001). Sex determination and estimation of stature from the long bones of the arm. Forensic Sci. Int..

[26] Kazama K, Kobayashi K, Sakamoto M (2016). In vivo three-dimensional analysis of distal radioulnar joint kinematics during forearm pronation-supination. J. Biomech. Sci. Eng..

[27] Besl P, McKay ND (1992). A method for registration of 3-D shapes. IEEE Trans. Pattern Anal. Mach. Intell..

[28] Fu E (2009). Elbow Position Affects Distal Radioulnar Joint Kinematics. J. Hand Surg..

